# Chiral population analysis: a real space visualization of X-ray circular dichroism

**DOI:** 10.1039/d5sc04423e

**Published:** 2025-07-31

**Authors:** Victor M. Freixas, Jérémy R. Rouxel, Sergei Tretiak, Niranjan Govind, Shaul Mukamel

**Affiliations:** a Department of Chemistry, Department of Physics and Astronomy, University of California Irvine 92697 USA vfreixas@uci.edu; b Chemical Sciences and Engineering, Argonne National Laboratory Lemont Illinois 60439 USA; c Physics and Chemistry of Materials, Theoretical Division, Los Alamos National Laboratory Los Alamos NM 87545 USA; d Center for Integrated Nanotechnologies, Los Alamos National Laboratory Los Alamos NM 87545 USA; e Physical and Computational Sciences Directorate, Pacific Northwest National Laboratory Richland WA 99352 USA; f Department of Chemistry, University of Washington Seattle Washington 98195 USA

## Abstract

The microscopic understanding of probing and controlling molecular chirality is of considerable interest. Numerous spectroscopic techniques are capable of monitoring molecular asymmetry and its consequences, ranging from the infrared to the X-ray regime. Resonant X-rays have long been used to investigate local atomic sites within molecules thanks to the localized nature of core electronic transitions. These techniques can be used to determine the extent to which chirality is a local *versus* a delocalized property. However, how to systematically partition dichroic contributions from the point of view of electronic structure simulations remains an open question. Here, we introduce the concept of chiral population analysis that connects chirality to the atomic orbital picture. In analogy with Mulliken population analysis, which assigns charges to atomic orbitals, chiral populations allow the dichroic response to be distributed among the participating atomic orbitals. This decomposition can be further visualized in real space by representing it in terms of isosurface plots, providing an intuitive way to connect the dichroic response to its origins. Thus chiral population analysis can be particularly useful to assess the extent to which a given electronic transition is sensitive to chirality as a local or global feature of the molecular geometry.

## Abstract 2

Chiral population analysis connects chiral response to the atomic orbital picture. Electric dipole induced displacements and magnetic dipole induced circulations manifest as lobes and loops. Spiral structures show both the electric and magnetic dipoles.

## Graphical abstract

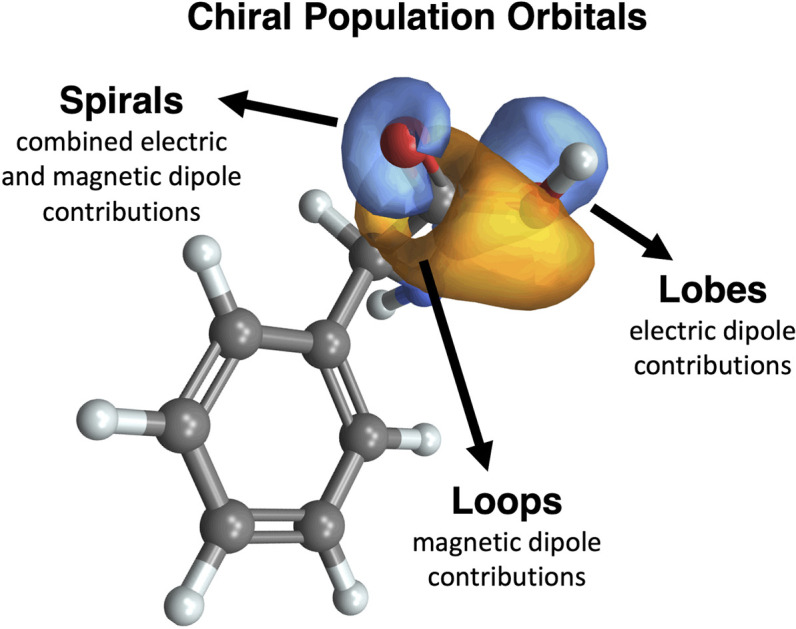

## Introduction

Quantum chemical techniques, such as the Mulliken population analysis^1–3^ and atomic orbital representations,^4,5^ have played a crucial role in converting the complex output of quantum chemistry calculations into chemically intuitive insights. These tools have allowed rationalizing chemical bonding theory and interpreting spectroscopic observables, thereby bridging computational results with conceptual understanding. In stereochemistry, chirality that results from the handedness of a molecular structure is usually discussed through a single scalar observable, the rotatory strength. This parameter measures the strength of a transition in circular dichroism (CD) or optical rotatory dispersion (ORD) spectra.^6–11^ However, it does not provide information on the spatial distribution of the chiral molecular response.

Recent developments in chirality-sensitive methods involving core-levels allow addressing how the chiral response is distributed within molecules.^12–18^ Furthermore, the time-dependent chiral response can be used to probe asymmetric nuclear motions.^19^ We have recently shown how X-ray CD (XCD) can be used to obtain information on the time-dependent local chirality of a helicene molecule undergoing a racemization process, thus providing dynamical insight into chiral transformations at the atomic level.^20^ While these studies show how different atomic sites have varying sensitivity to molecular chirality, a direct connection between the dichroic response and the atomic orbital picture is not fully established. In this contribution, we close this gap connecting the chiral response to the atomic orbital framework. We introduce chiral population analysis (CPA) as a novel chemical analysis method allowing the real space visualization of the chiral response.

The paper is structured as follows. We begin by outlining the theoretical framework used to compute CD signals in terms of molecular properties accessible from standard quantum chemistry software. Next, we introduce the concept of chiral populations, which decompose the CD response into atomic orbital contributions. We then demonstrate how CPA can qualitatively identify the electric, magnetic, or combined origins of the chiral contributions of the second excited state of the hydroxyl oxygen K-edge manifold for the phenylglycine molecule. This is followed by an application of CPA across different energy ranges to capture the interplay between two distinct chiral sources along the isomerization pathway of an azobenzene–phenylglycine (APgly) molecule.^21^ Finally we conclude with a summary and perspectives on future applications of the chiral populations for visualizing and analyzing electronic structure and its spectroscopic signatures.

## Results and discussion

The primary quantity characterizing the molecular chirality measured by linear spectroscopic signals (CD or ORD) is the rotatory strength defined by1
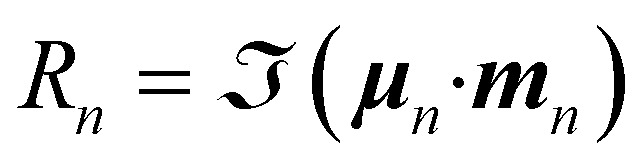
where ***μ***_*n*_ and ***m***_*n*_ are, respectively, the electric and magnetic transition dipole moments between the molecular ground state and excited state |*n*〉 with transition energy *E*_*n*_. The rotatory strength measures the chirality of a transition between two electronic states and can be used to compute the CD or ORD signals through sum-over-states expressions, see SI Section 1.

Using orbital isosurfaces to represent electronic structure properties is an intuitive and convenient way to understand the shape and character of electron distributions in molecules. The approach involves calculating a scalar field given by a superposition of atomic orbitals (AOs), which is usually visualized as an isosurface. This representation depends on the coefficients that weight each AO. Common examples are molecular orbitals (MOs),^22^ where the same coefficients defining the MO in terms of the AO are used as the AO weights.

For excited electronic states, two common isosurface plots can be generated from the one-body reduced transition density matrix (TDM). Its diagonal terms represent the transition charge density associated with the electronic excitation.^23^ Alternatively, natural transition orbitals can be computed by a singular value decomposition (SVD) of the TDM. They can be used to analyze charge transfer states to investigate the departure and arrival orbitals of the excited electron.^24–27^

Using a scalar field for representing the rotatory strength contributions in real space was suggested by Nafie^28^ and later by Fusè *et al.* for vibrational circular dichroism.^29^ In their approach, two different scalar fields are defined as projections onto each other of the electric and magnetic contributions to the vibrational transition current density. Here, we partition the contributions to the chiral response directly in the AO basis instead. The rotatory strength in eqn (1) can be written as (see the SI for detailed derivation):2
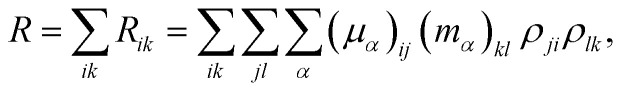
where the sums over *i*, *j*, *k*, *l* run over the AO basis set, (*μ*_*α*_)_*ij*_ and (*m*_*α*_)_*kl*_ are the electric and magnetic dipole moment matrices along the spatial direction *α*, respectively, and *ρ*_*ij*_ and *ρ*_*kl*_ are TDMs. Here all matrices are given in the AO representation. *R*_*ik*_ can be arranged in a matrix ***R*** that represents the contributions to the rotatory strength from the electric and magnetic dipole couplings from AO *i* and *k*, respectively. The matrix ***R*** can be represented in real space in a number of waysusing isosurface plots. For instance, using its diagonal terms would lead to a representation of the orbitals contributing with both their electric and magnetic dipoles, in analogy to the electronic states representation by the diagonal elements of the TDM.

We construct a representation of the chirality following an analogy with the Mulliken population analysis for electron densities.^1,2^ To get a compact picture of the AO contributions to the chiral response, the ***R*** matrix can be recast as a combination of antisymmetric and a symmetric matrices:

3 ***R*** = ***R***^(asym)^ + ***R***^(sym)^,

4
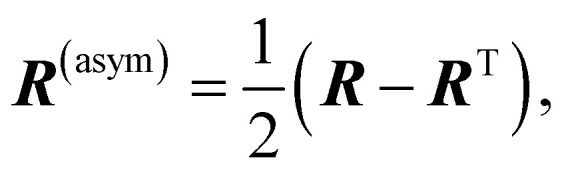
5
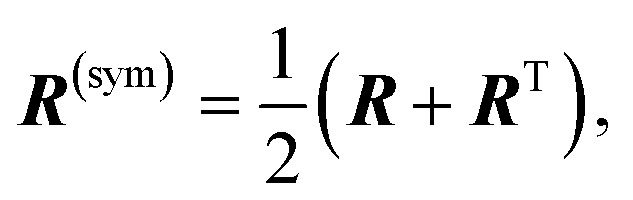
where ***R***^T^ stands for the transpose of matrix ***R***. The antisymmetric matrix does not contribute to the rotatory strength. In contrast, the symmetric component has cancellation contributions coming from atom pairs, while retaining the physically meaningful constructive couplings that generate the chiral response. We define a chiral population *R*^(AO)^_*i*_ based on the symmetrized chiral coupling matrix ***R***^(sym)^:6
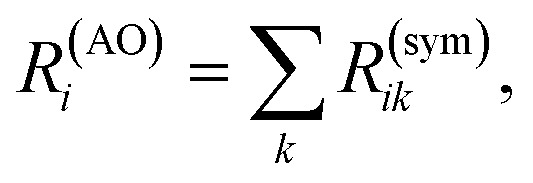


Unlike Mulliken population analysis, which requires the inclusion of AO overlaps to recover the total electron density, the chiral population summation over AO contributions yields the total rotatory strength directly.

Chiral populations can be further projected into real space by constructing a three-dimensional scalar field, defined as a linear combination of AOs weighted by their corresponding *R*^(AO)^_*i*_ values. We refer to the resulting spatial functions *Φ*(***r***) as chiral population orbitals, being the key quantity of this study and defined as:7
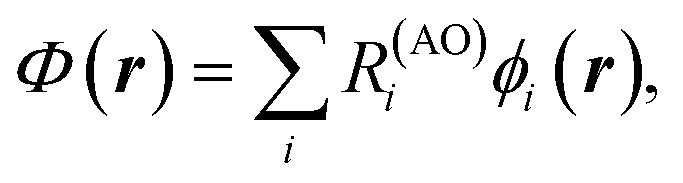
where *ϕ*_*i*_(***r***) is the *i*th atomic orbital. Note that the non-orthogonality of the AO basis does not preserve the additive property of the original chiral decomposition. Löwdin-orthogonalized AOs or molecular orbitals can restore additivity (the spatial integration of the chiral population orbitals reduces to the rotatory strengths). However, this orthogonalization is not essential in the construction of intuitive and chemically meaningful representations. The chiral population orbitals serve as an insightful map of the local constructive and destructive interferences between chiral-active orbitals, highlighting regions of dominant electric, magnetic, or mixed contributions. This is a much more detailed representation compared to a single scalar observable.

Even in molecules that are planar or do not possess intrinsic chiral structural elements, local chiral populations can still arise in electronic transitions. In these cases, the overall molecular symmetry enforces an exact cancelation of these contributions, resulting in a vanishing total rotatory strength. This implies that the CPA can reveal hidden local chiral fingerprints that, while individually significant, mutually cancel out in achiral systems.

### CPA on phenylglycine

**Fig. 1:**
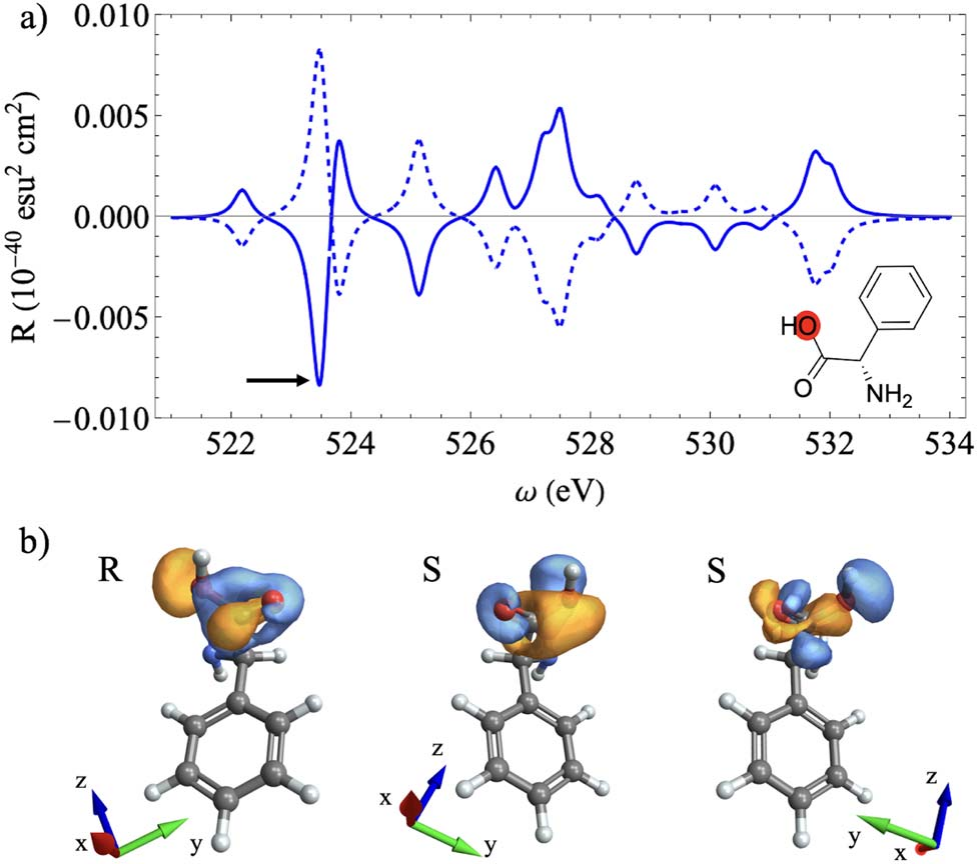
(a) XCD spectra in the range of the hydroxyl oxygen K-edge for the *R* (solid lines) and *S* (dashed lines) enantiomers of the phenylglycine molecule. The inset on the right bottom shows the molecular structure. The hydroxyl oxygen is marked in red. The arrow shows the second transition of the manifold. (b) Chiral population orbitals corresponding to the second transition of the manifold. The R and S labels correspond to the Cahn–Ingold–Prelog rules.^35^

We now discuss the CPA for two molecular systems. First, we illustrate the qualitative capabilities of the CPA for a simple known chiral molecule, phenylglycine, shown in Fig. 1a. Here, several atomic K-edges can be resolved, such as the carbonyl and hydroxyl oxygens within the glycine moiety carboxyl group.^30^ This is the case for X-ray absorption when specific atoms appear under different bonding conditions.^31–33^ As such, XCD signals at multiple X-ray chromophores provide multidimensional insight into chiral features.^34^

Fig. 1 shows the simulated XCD spectra at the hydroxyl oxygen K-edge for both enantiomers of phenylglycine as solid and dashed lines, respectively. While additional chiral population orbitals for various K-edges are provided in the SI, our analysis here focuses on the second transition of the hydroxyl oxygen K-edge, as indicated by the arrow in Fig. 1a. The corresponding chiral population orbitals are shown in Fig. 1b. These orbital representations have three distinct features that allow one to trace the origin of the chiral response. Lobe features, such as the ones around the hydroxyl oxygen atom, represent electronic displacements induced by electric transition dipoles contributing to the dichroic response. In contrast, loop features, like those around the carboxyl carbon, represent electronic circulation induced by magnetic transition moments. Finally, spiral-like structures, such as the one over the carbonyl oxygen in the left and middle panels of Fig. 1b, correspond to spatial regions where both the electric and magnetic dipole contributions play a significant role in shaping the dichroic response.

Fig. 1b illustrates the orientation dependence of the CPA representation. The middle and right panels correspond to the same enantiomer, but with different orientations in their respective systems of coordinates. The molecule in the right panel is rotated 180° around the *z*-axis with respect to the one in the middle panel, and the projections are aligned for easier comparison. We can notice how the choice of the coordinate system orientation alters the representation, reflecting how the projections of the electric and magnetic dipoles onto each other contribute to the rotatory strength.^29^ Notably, this orientation dependence does not hinder the CPA. For instance, for these two orientation choices, we can notice that the carbonyl oxygen contributes through its electric dipole, while the hydroxyl oxygen contributes through both the electric and magnetic dipoles, indicated by the presence of lobes and spirals, respectively.

Other state contributions to the XCD signal can also be examined in detail. For instance, the first row and first column of Fig. S1b show the chiral population orbitals corresponding to the first transition of the hydroxyl oxygen K-edge manifold. This chiral population density consists mainly of two lobes with opposite phases located around the hydroxyl oxygen, from where the core electron is excited. This distribution indicates that the main contribution of this transition to the chiral response comes from electric dipole induced electronic displacements. In contrast, the third transition of the manifold shows a vanishing dichroic response. The corresponding chiral population orbitals vanish, as shown in the first row and third column of Fig. S1b. Notably, a vanishing dichroic response does not necessarily imply that the chiral population orbitals themselves vanish, as relevant local contributions of chiral populations could cancel each other by symmetry, rendering no circular dichroism. However, the converse does hold, a vanishing chiral population implies a vanishing rotatory strength, as in the case here.

### CPA on fluorinated azobenzene–phenylglycine

**Fig. 2:**
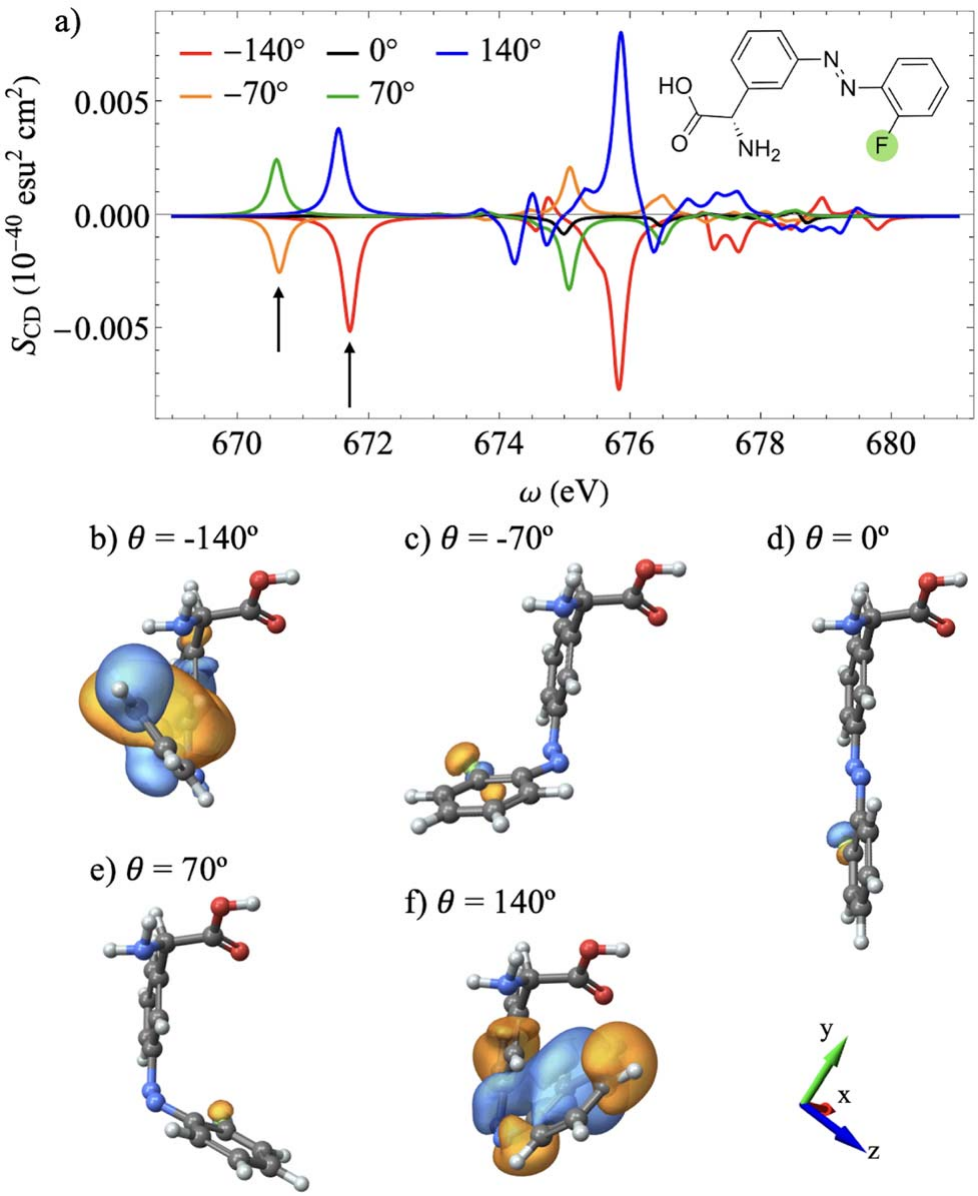
(a) XCD spectra in the range of the fluorine K-edge for different azobenzene dihedral angles. (b–f) Chiral population orbitals for the transitions depicted by the black arrows (S_1_) scanned across the different dihedral angles. The orientation of the system of coordinates is shown in (d).

We next turn to the more structurally complex fluorinated azobenzene–phenylglycine (APgly) molecule,^21^ depicted in the inset of Fig. 2. The phenylglycine moiety has a chiral center that serves as a source of static and local chirality. Azobenzene is a very well-known photoswitch that isomerizes upon photoexcitation.^36,37^ Each isomerization path carries a distinct chiral response, making the azobenzene moiety a source of dynamic chirality. A fluorine atom is introduced near the azo group to serve as an X-ray chromophore. To explore the isomerization process, we scan the CNNC dihedral angle *θ*, using the *trans*-azobenzene planar conformation as a reference (*θ* = 0°), from −140° to 140°. This is a simplified model that spans two possible *trans*-to-*cis* isomerization pathways.

XCD proves particularly valuable in this context, as different absorption edges can be selectively sensitive to distinct chiral features along the azobenzene isomerization pathway. In the following we use CPA to illustrate three representative cases. First, we present an example of a core electronic transition that is sensitive exclusively to the azobenzene isomerization. Second, we show an example of a core electronic transition that is only sensitive to the phenylglycine chiral center. Finally, we highlight an example of a chiral transition in the dichroic response resulting from a core electronic transition changing from being sensitive to the phenylglycine chiral center dichroism, to being sensitive to the azobenzene global chirality. CPA provides a powerful interpretative tool in this context as it enables a direct connection between chiral spectral features and their AO origins.

Fig. 2a shows the XCD spectra at the F K-edge for APgly for different *θ* angles. The antisymmetry of the XCD signal for configurations with *θ* = −140° and *θ* = 140°, and for configurations with *θ* = −70° and *θ* = 70°, indicates that this spectral range is sensitive to the azobenzene dynamic chirality. This is further confirmed by the vanishing CD signal for *θ* = 0°, where the azobenzene moiety is in a planar configuration.

The CPA provides a direct connection to the AO picture, as it can be shown by examining the chiral population orbital isosurface plots corresponding to the first excitation of the manifold across the dihedral scan. These orbitals for *θ* = −70°, *θ* = 0°, and *θ* = 70° are shown in Fig. 2c–e, respectively. The small lobe features over the F atom indicate a weak chiral response dominated by the electric transition dipole over the F atom. In particular, for *θ* = 0°, the electric dipole contributions to the chiral response align with the ring plane, further quenching out the dichroic response. This is a consequence of the symmetry constraint imposed by the planar configuration. Moreover, for Fig. 2c and e, the lobe phases that represent the electric dipole contributions are opposite, resulting in inverted dichroic responses. Finally, Fig. 2b and f show the corresponding orbitals for *θ* = −140° and *θ* = 140°, respectively. Here, the contributions extend through space toward the phenylglycine ring due to its spatial proximity, but only to the ring and not to the glycine moiety. This through-space extension to the neighboring ring enhances the chiral response, as shown in Fig. 2a, which is consistent with more extended isosurfaces.

**Fig. 3:**
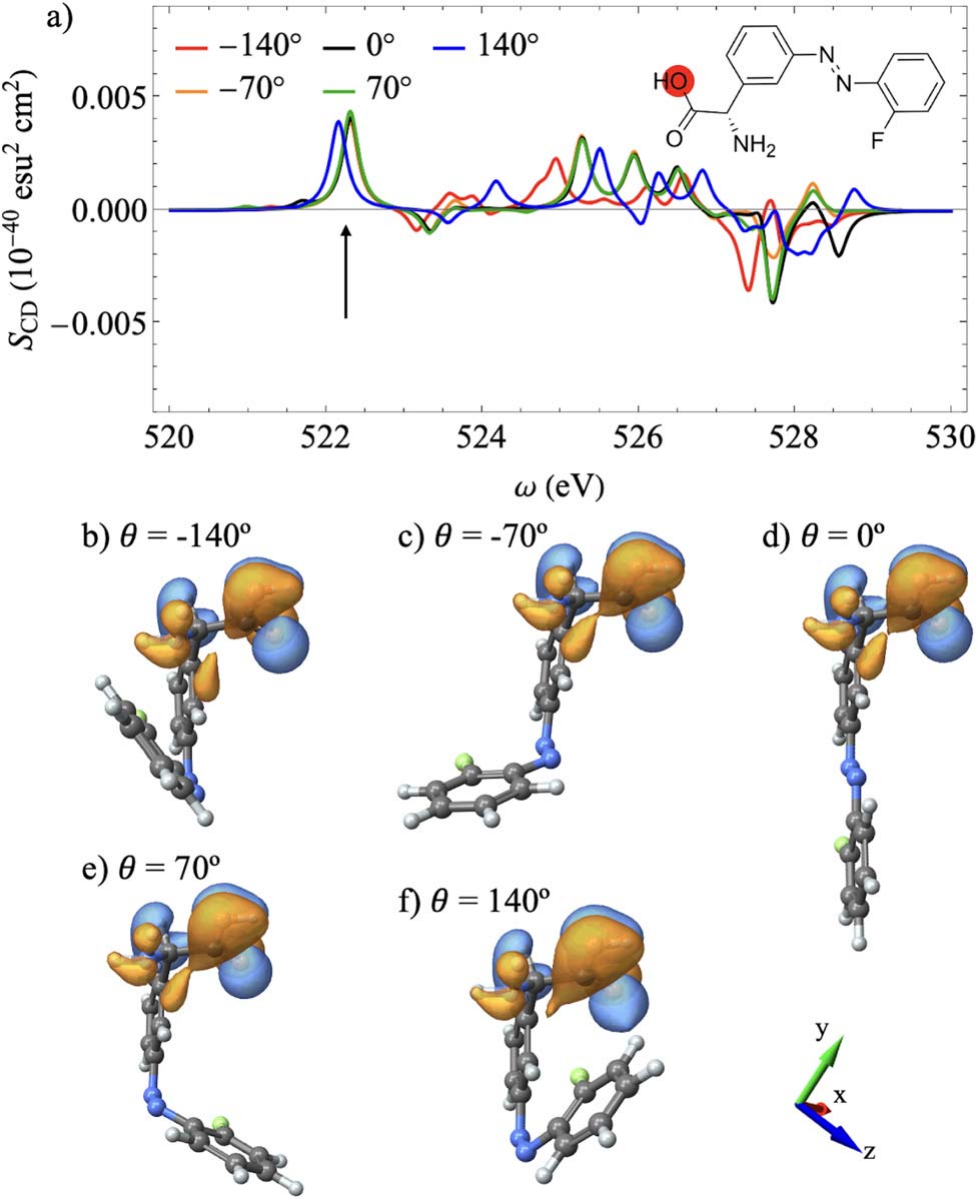
(a) XCD spectra in the range of the carbonyl oxygen K-edge for different azobenzene dihedral angles. (b–f) Chiral population orbitals for the transitions depicted by the black arrow (S_2_) scanned across the different dihedral angles. The orientation of the system of coordinates is shown in (d).

Fig. 3a depicts the XCD spectra at the hydroxyl oxygen K-edge for different dihedral angles. In this spectral range, variations in the azobenzene dihedral angle induce mainly energy shifts of the XCD spectra peaks, without causing inversions in the dichroic signal. This relatively static behavior suggests that the core electronic transitions in this range are not sensitive to the dynamic chirality of azobenzene. Fig. 3b–f show the chiral population orbitals corresponding to the second core-excited state across the dihedral scan denoted by the arrow in the spectra. The invariance of these peaks with respect to the azobenzene dihedral angle, as shown in Fig. 1a, indicates that this spectral range is sensitive only to the static local chirality arising from the phenylglycine chiral center. This is further confirmed from the corresponding orbital plots in Fig. 3b–f, which show that only AOs on the glycine moiety contribute to the chiral response. Furthermore, the corresponding isosurface plots remain approximately invariant across the dihedral angle scan.

**Fig. 4:**
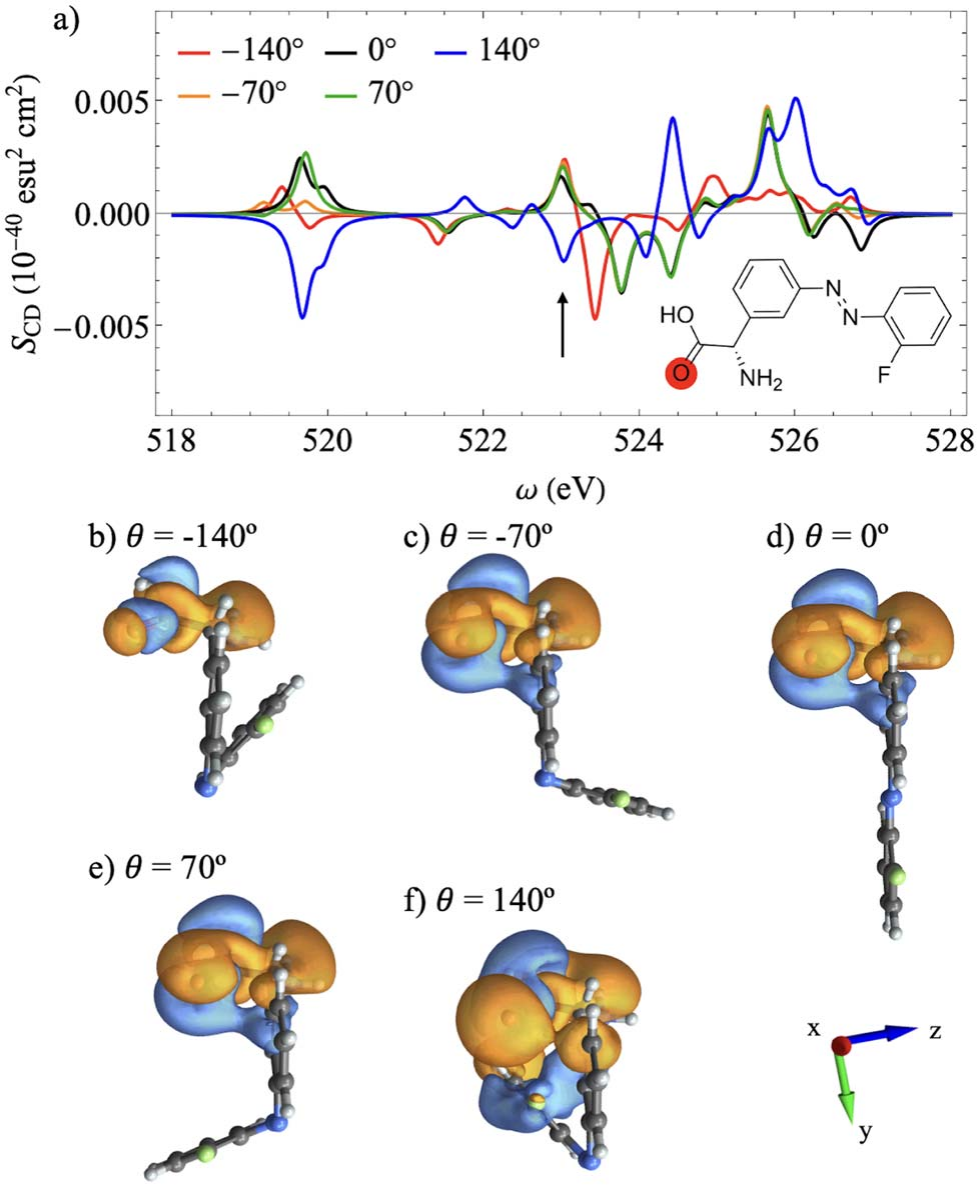
(a) XCD spectra in the range of the hydroxyl oxygen K-edge for different azobenzene dihedral angles. (b–f) Chiral population orbitals for the transitions depicted by the black arrow scanned across the different dihedral angles. The orientation of the system of coordinates is shown in (d).

Fig. 4a shows the XCD spectra at the carboxyl O K-edge at different dihedral angles. We observe that most peaks retain their intensity, sign and energy for dihedral angles ranging from *θ* = −70° to *θ* = 70°, indicating that, within this range, the XCD response is primarily sensitive to the chiral center static chirality. However, at larger dihedral angles, *i e. θ* = ±170°, several peak changes can be noticed. This indicates that the corresponding core electronic transitions become increasingly sensitive to the azobenzene global chirality. Fig. 4b, c and e show the chiral population orbitals corresponding to the sixth excitation of the core-excited state manifold, while Fig. 4d and f show the orbitals corresponding to the seventh excitation of the manifold. These excitations have the same wavefunction character because of a state crossing in this region of the nuclear conformational space, where the sixth and seventh excited states of the manifold switch order. The states contributing to the peaks denoted by the arrow in Fig. 4a maintain a consistent electronic distribution across the dihedral angle scan. The chiral response remains similar from *θ* = −140° to *θ* = 70°, which corresponds to similar orbitals as shown in Fig. 4b–e. However, the chiral response changes dramatically at *θ* = 140°. This change can be rationalized by examining Fig. 4f, which shows the chiral population orbital extending to the opposite ring.

Although our CPA focused on the chiral response of individual structures, in real molecular systems, several conformations can contribute to the CD spectra. For the *trans*–*cis* azobenzene isomerization, two different pathways lead to two possible distinct *cis* conformations. The chiral response of such a racemic mixture would vanish, so we can add an additional chiral source to the molecule to break the chiral symmetry. The CPA of the matrix resulting from averaging ***R***^(sym)^ over the ensemble should thus be useful to rule out the AO contributions that cancel out within the ensemble. Connecting the averaged matrix to a real space isosurface plot is not straightforward as racemic mixtures typically lack a unique representative molecular structure. Nevertheless, CPA will be very useful in future studies of molecular chirality with more or less delocalized nuclear wavepackets.

## Conclusions

Conventional circular dichroism analyses reduce each electronic transition to a single scalar quantity, the rotatory strength. Therefore, circular dichroism spectra contain no internal chemical resolution. The goal of the present work is to decompose the same rotatory strength into a chemically meaningful distribution of contributions over AOs, which can be further projected into real space. In order to do so, we have introduced CPA as a novel quantum chemical analysis method, analogous to Mulliken population analysis for charge distributions. While Mulliken charges quantify the distribution of electronic density over AOs, CPA partitions the molecular dichroic response among AOs. The resulting chiral population orbitals offer spatial insight into how chirality is expressed across a molecule. This strategy provides two advantages. First, it provides a visually intuitive representation of where chirality originates within a molecule. Second, it allows to distinguish whether a given transition probes chirality as a local or global feature. Given that CPA is defined from the transition density matrices and dipole moment integrals, it is intentionally richer than, and not derivable from, the XCD spectra alone.

In CPA visualizations, electric transition dipole contributions appear as lobe-like features, magnetic dipole contributions as loop structures, and regions with both components give rise to spiral patterns. CPA relies solely on the transition density matrix and properties of the AO basis, making it straightforward to implement using standard quantum chemistry software. It can also be extended to transitions between excited states and to monitor time-dependent chiral responses.

This spatially resolved picture of chirality provides a powerful framework for interpreting and designing enantioselective processes, including asymmetric catalysis^38–40^ and photocatalysis.^41^ It can guide the optimization of chiral ligands by identifying regions of strong rotatory strength and supports the rational design of X-ray chromophores for resonant X-ray circular dichroism experiments,^15^ where localized transitions enable site-specific chirality measurements.

## Computational details

The electronic structure computations, including X-ray computations,^42,43^ presented in this work were performed with the NWChem quantum chemistry software,^44^ at the time-dependent density functional level with the PBE0 functional^45^ and the cc-pVDZ basis set.^46^ This combination has shown a good agreement with higher levels of electronic structure computations for azobenzene.^47^ The velocity gauge was used for the computation of the transition electric dipoles to remove the dependence of the rotatory strength on the origin of coordinates. Cube files were generated with the help of the Multiwfn analysis toolbox.^48^ To generate the dihedral angle scan, the initial *trans* structure was aligned to the principal axis of its inertia tensor. The lighter ring was then rotated clockwise and counterclockwise around the azo bond. Isovalues of 1.5 × 10^−6^ a.u. were used for the isosurface representations.

## Author contributions

S. M., N. G., S. T, and J. R. R. designed the project. V. M. F. deduced the CPA equations, performed the simulations and prepared the figures. All authors analyzed and interpreted the results. The manuscript was written with contributions from all authors.

## Conflicts of interest

The authors declare no conflict of interest.

## Supplementary Material

SC-016-D5SC04423E-s001

## Data Availability

The data supporting this article have been included as part of the SI: Optical rotation and circular dichroism; partition of the rotatory strength. See DOI: https://doi.org/10.1039/d5sc04423e.
