# Supplementary material for: Chiral population analysis: a real space visualization of X-ray circular dichroism
Source: Chem Sci. 2025 Jul 31;16(35):16218–24. doi: 10.1039/d5sc04423e (PMC12343753; doi:10.1039/d5sc04423e)
Supplement: SC-016-D5SC04423E-s001 [file SC-016-D5SC04423E-s001.pdf]

Supplementary Information (SI) for Chemical Science.
This journal is © The Royal Society of Chemistry 2025

SUPPLEMENTARY INFORMATION
Chiral population analysis: A real space visualization of X-ray circular dichroism

Victor M. Freixas[1,*], Jérémy R. Rouxel[2], Sergei Tretiak[3,4], Niranjan Govind[5,6], and Shaul Mukamel[1]

[1]Department of Chemistry and Department of Physics and Astronomy, University of California, Irvine, 92697, USA
[2]Chemical Sciences and Engineering Division, Argonne National Laboratory, Lemont, Illinois 60439, United States
[3]Physics and Chemistry of Materials, Theoretical Division, Los Alamos National Laboratory, Los Alamos, NM 87545, USA.
[4]Center for Integrated Nanotechnologies, Los Alamos National Laboratory, Los Alamos, NM 87545, USA.
[5]Physical and Computational Sciences Directorate, Pacific Northwest National Laboratory, Richland, WA 99352
[6]Department of Chemistry, University of Washington, Seattle, WA 98195, USA

July 14, 2025

Contents

1 Optical Rotation and Circular Dichroism 1
2 Partition of the Rotatory Strength 2
3 Chiral population orbitals of phenylglycine across different K-edges 2

1 Optical Rotation and Circular Dichroism

Optical rotation $\Delta\theta$ and circular dichroism η signals can be determined by the dispersive and absorptive parts of the optical activity tensors[1]. After averaging over all the possible orientations of the molecule in an isotropic medium, only the diagonal elements of the electric dipole - magnetic dipole frequency dependent tensor $G'_{\alpha\alpha}(\omega)$ survive:

$$G'_{\alpha\alpha}(\omega) = -\frac{2}{\hbar}\sum_{j\neq n}\frac{\omega_{jn}^2-\omega+i\omega\Gamma_{nj}}{\left(\omega_{jn}^2-\omega\right)^2+\omega^2\Gamma_{nj}^2}\Im\left[\left(\langle n|\hat{\mu}_\alpha|j\rangle\langle j|\hat{m}_\alpha|n\rangle\right)\right], \quad (1)$$

where α refers to the spatial directions in real space, $\hbar$ is the reduced Planck constant, $|n\rangle$ and $|j\rangle$ are the initial and final states of the transitions, respectively, ω_{jn} is the transition frequency, Γ_{jn} is a phenomenological damping factor, $\Im$ refers to the imaginary part, and $\hat{\mu}_\alpha$ and $\hat{m}_\alpha$ are the electric and magnetic dipole operators, respectively. Optical rotation $\Delta\theta$ and circular dichroism η signals can then be written as[1]:

$$\Delta\theta(\omega) = -\frac{1}{3}\omega\mu_0 lN\sum_{\alpha\in(x,y,z)}\Re\left[G'_{\alpha\alpha}(\omega)\right], \quad (2)$$

$$\eta(\omega) = -\frac{1}{3}\omega\mu_0 lN\sum_{\alpha\in(x,y,z)}\Im\left[G'_{\alpha\alpha}(\omega)\right], \quad (3)$$

where μ_0 is the vacuum permeability, l is the length path through the sample, N is the number density of chiral molecules, and $\Re$ refers to the real part. Both the optical rotation and the circular dicrhroism signals depend on the imaginary part of the inner product between the electric and magnetic dipoles for a given transition, which is usually named the rotatory strength $R_{n\to j}$:

$$R_{n\to j} = \sum_{\alpha\in(x,y,z)} \Im\left[\left(\langle n|\hat{\mu}_\alpha|j\rangle\langle j|\hat{m}_\alpha|n\rangle\right)\right]. \tag{4}$$

2 Partition of the Rotatory Strength

In the following, in order to ease the notation, we will work with the imaginary part of the magnetic transition dipole moment and will drop the dependence on the specific transition $n \to j$. The component $\alpha = (x, y, z)$ of the electric transition dipole can be written as:

$$\mu_\alpha = \mathrm{Tr}\left[\boldsymbol{\mu}_\alpha \rho\right], \tag{5}$$

where Tr stands for trace and ρ is the TDM of the corresponding transition, which can be written in terms of creation $c_j^\dagger$ and annihilation c_i operators over AOs[2].

The bold font of $\boldsymbol{\mu}_\alpha$ in equation 5 denotes its matrix character:

$$(\boldsymbol{\mu}_\alpha)_{ij} = \langle\chi_i|\hat{\mu}_\alpha|\chi_j\rangle, \tag{6}$$

where χ_i and χ_j are two elements of the AO basis. Similarly, for the magnetic transition dipole, we have:

$$m_\alpha = \mathrm{Tr}\left[\boldsymbol{m}_\alpha \rho\right], \tag{7}$$

with:

$$(\boldsymbol{m}_\alpha)_{ij} = \langle\chi_i|\hat{m}_\alpha|\chi_j\rangle, \tag{8}$$

The rotatory strength can be written directly in the AO basis as:

$$R = \sum_{ik} R_{ik}, \tag{9}$$

where:

$$R_{ik} = \sum_{jl}\left((\boldsymbol{\mu}_x)_{ij}(\boldsymbol{m}_x)_{kl} + (\boldsymbol{\mu}_y)_{ij}(\boldsymbol{m}_y)_{kl} + (\boldsymbol{\mu}_z)_{ij}(\boldsymbol{m}_z)_{kl}\right)\rho_{ji}\rho_{lk}, \tag{10}$$

Here, the indexes j and l come from the matrix multiplications within the traces in equations 5 and 7. The terms R_{ik} can be arranged in a matrix $\boldsymbol{R}$ that represents the contributions to the rotatory strength from the electric and magnetic dipole couplings from AO i and k, respectively.

3 Chiral population orbitals of phenylglycine across different K-edges

In this section we display the XCD spectra for phenylglycine at different K-edges. The first 20 chiral population orbitals are also shown as a table of graphics. In the first column we can find the first five isosurface plots for the corresponding manifold, in the second column we can find the next five, and so on.

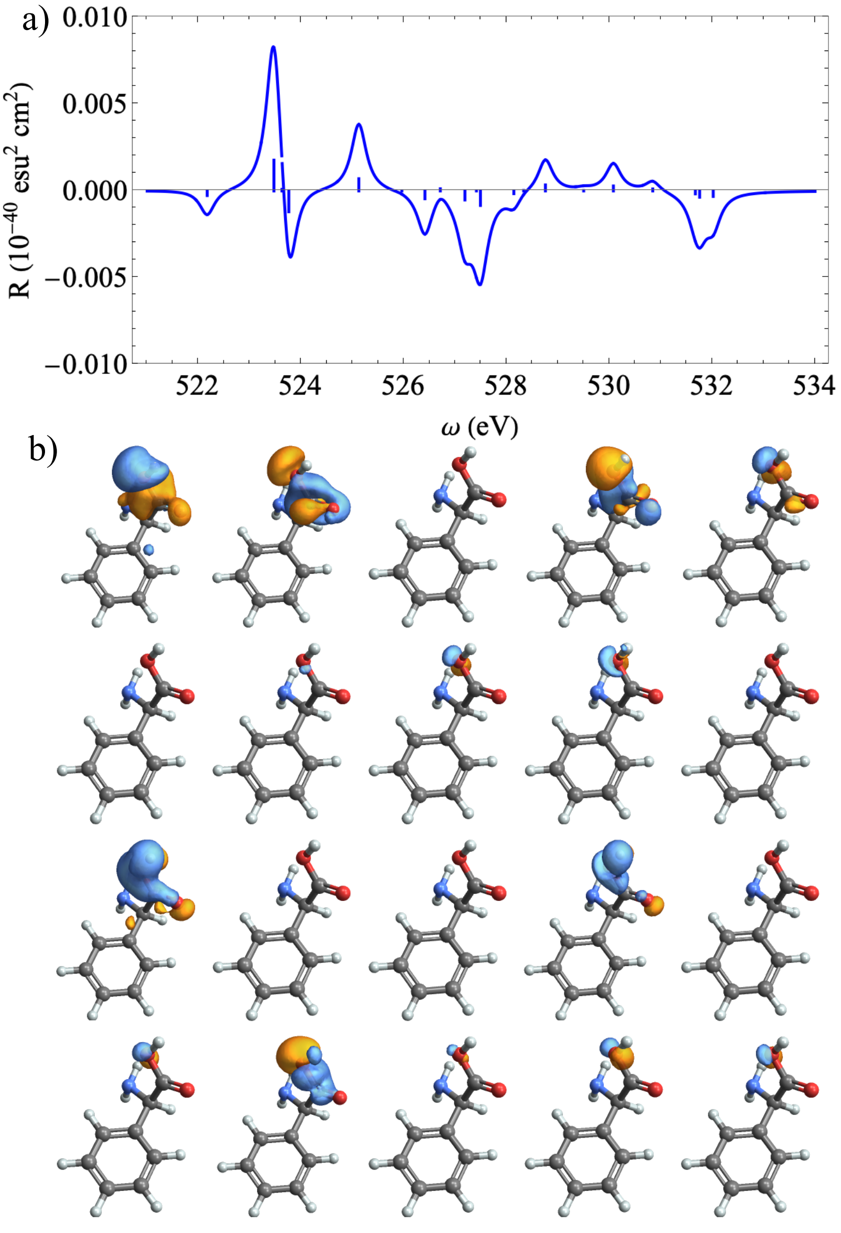

Figure S1: a) XCD spectra in the range of the hydroxyl oxygen K-edge for phenylglycine. Vertical sticks show the rotatory strengths. b) Chiral population orbitals for the first 20 transitions of the manifold.

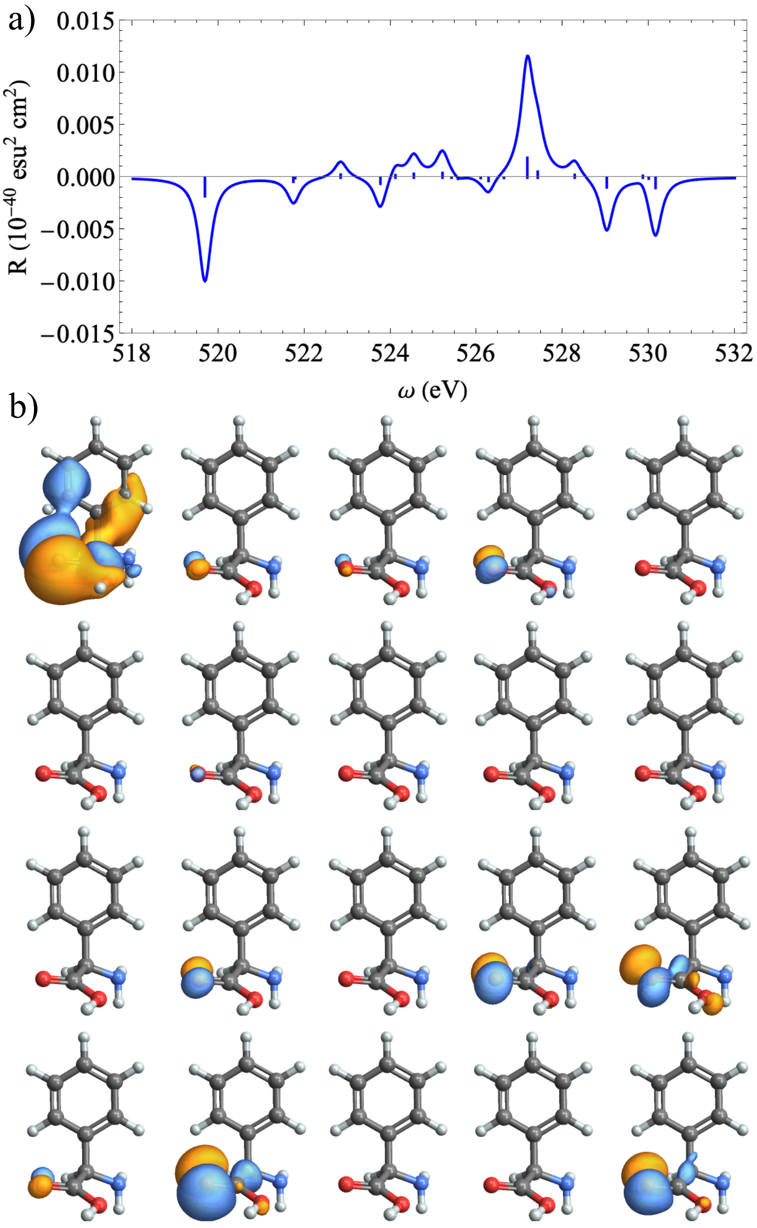

Figure S2: a) XCD spectra in the range of the carboxyl oxygen K-edge for phenylglycine. Vertical sticks show the rotatory strengths. b) Chiral population orbitals for the first 20 transitions of the manifold.

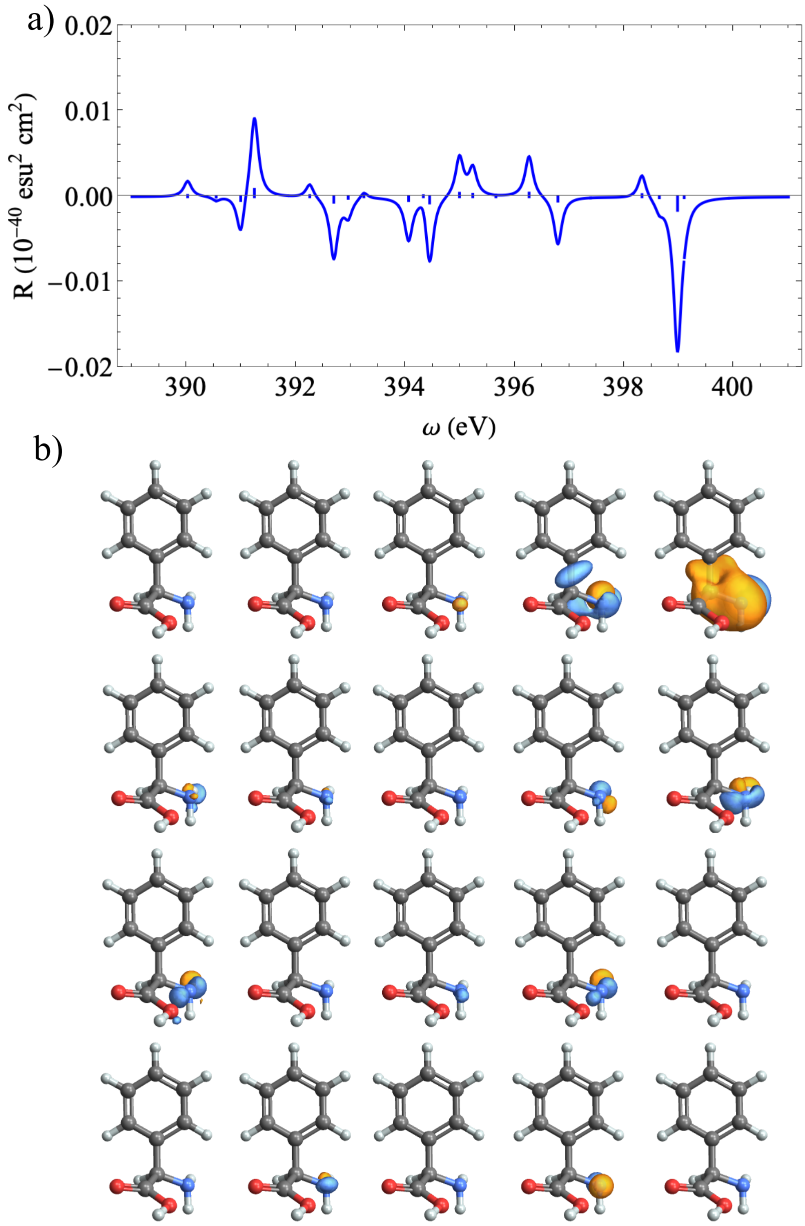

Figure S3: a) XCD spectra in the range of the nitrogen K-edge for phenylglycine. Vertical sticks show the rotatory strengths. b) Chiral population orbitals for the first 20 transitions of the manifold.

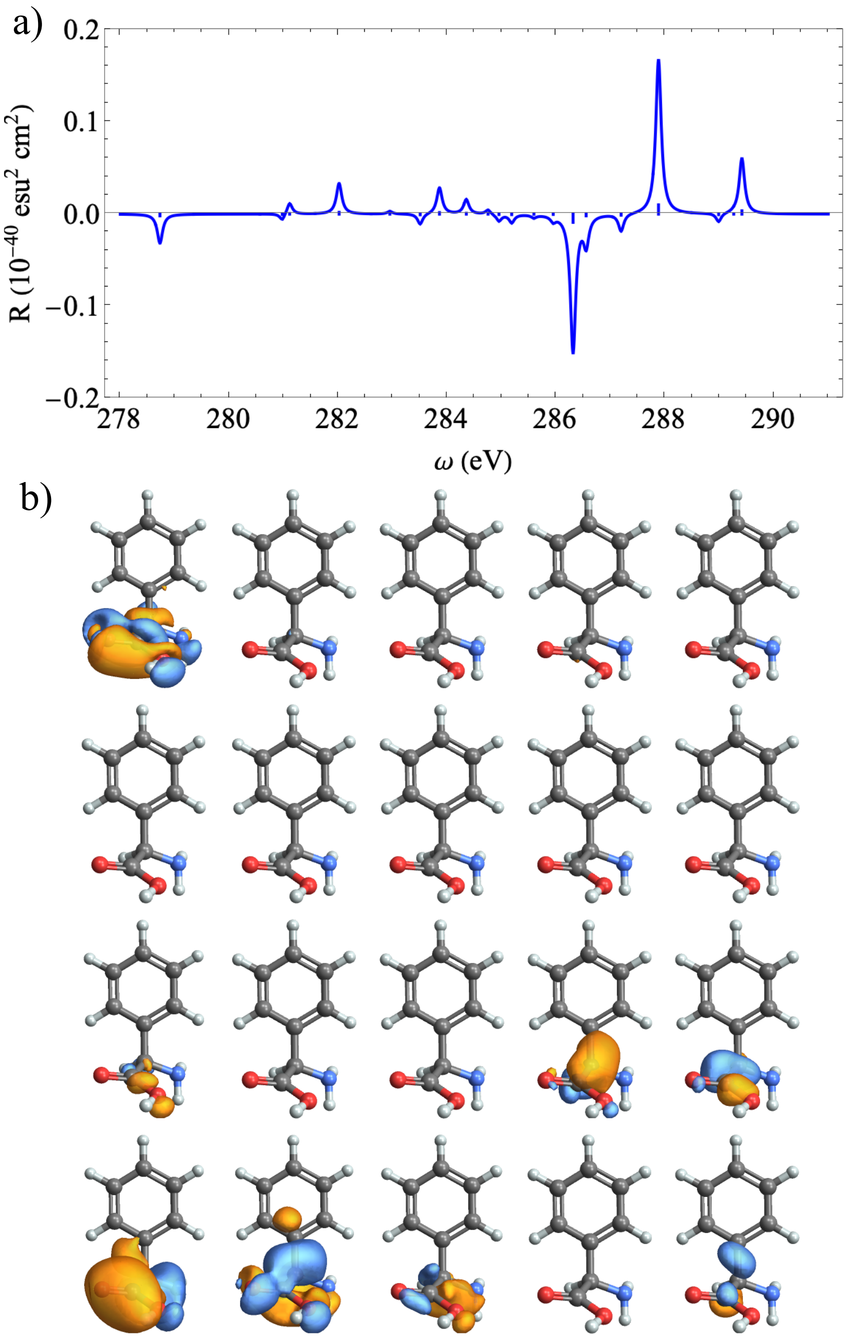

Figure S4: a) XCD spectra in the range of the carboxyl carbon K-edge for phenylglycine. Vertical sticks show the rotatory strengths. b) Chiral population orbitals for the first 20 transitions of the manifold.

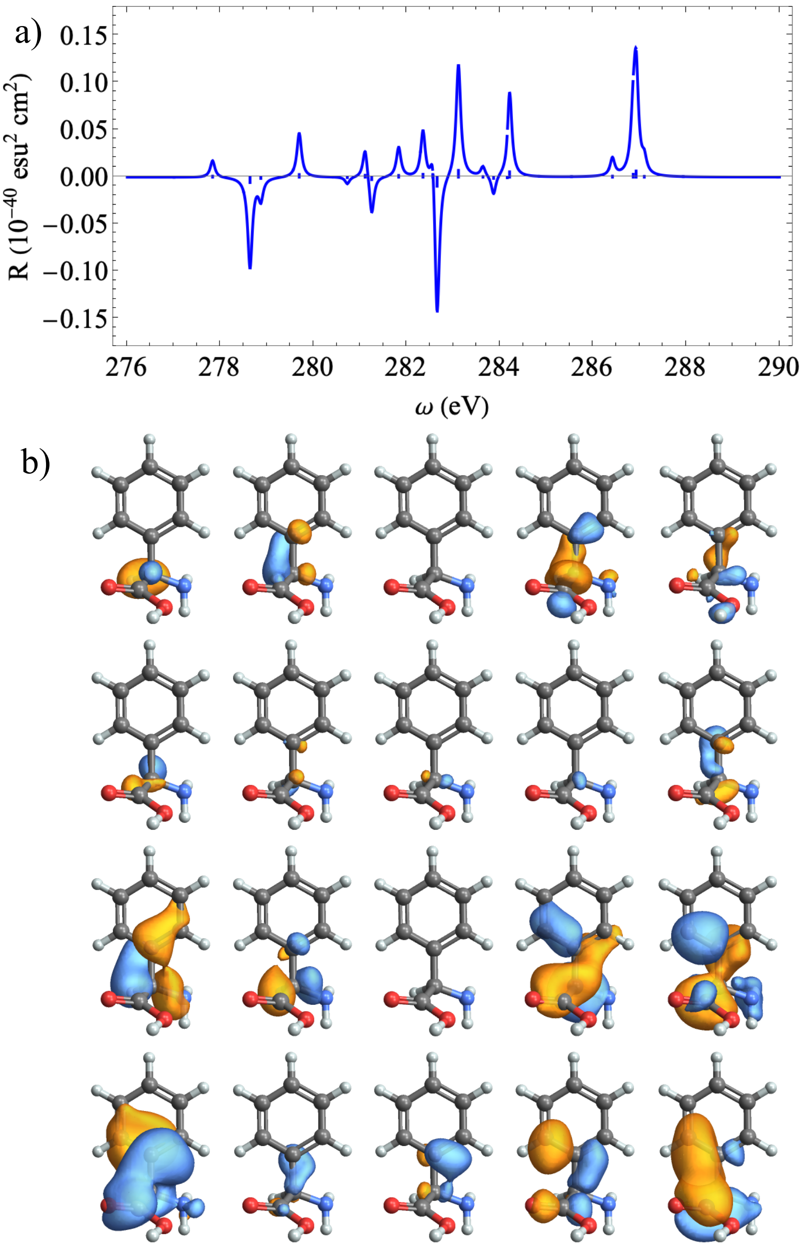

Figure S5: a) XCD spectra in the range of the chiral center carbon K-edge for phenylglycine. Vertical sticks show the rotatory strengths. b) Chiral population orbitals for the first 20 transitions of the manifold.

References

[1] Laurence D. Barron. *Molecular Light Scattering and Optical Activity*. Cambridge University Press, 2nd edition, 2004.

[2] Sergei Tretiak and Shaul Mukamel. Density matrix analysis and simulation of electronic excitations in conjugated and aggregated molecules. *Chemical reviews*, 102(9):3171–3212, 2002.
